# Transient photothermal inactivation of *Escherichia coli* stained with visible dyes by using a nanosecond pulsed laser

**DOI:** 10.1038/s41598-020-74714-5

**Published:** 2020-10-20

**Authors:** Yuji Kohmura, Natsuho Igami, Ichiro Tatsuno, Tadao Hasegawa, Takahiro Matsumoto

**Affiliations:** 1 Graduate School of Medical Sciences, Nagoya City University, Nagoya, 467-8601 Japan; 2 Graduate School of Design and Architecture, Nagoya City University, Nagoya, 464-0083 Japan; 3 Lucir Incorporated, Tsukuba, Ibaraki 300-2667 Japan

**Keywords:** Biophysics, Microbiology

## Abstract

Efficient inactivation of *Escherichia coli* (*E. coli*) under visible (532 nm) pulsed light irradiation was achieved by fusion of a visible light-absorbing dye with *E. coli*. Inactivation experiments showed that 3-log inactivation of *E. coli* was obtained within 20 min under a 50 kJ/cm^2^ dose. This treatment time and dose magnitude were 10 times faster and 100 times lower, respectively, than the values previously obtained by using a visible femtosecond laser. The mechanism of bacterial death was modeled based on a transient photothermal evaporation effect, where a quantitative evaluation of the temperature increase was given based on the heat transfer equation. As a result of this theoretical analysis, the maximum temperature of the bacteria was correlated with the absorption ratio, pulse energy, and surface-to-volume ratio. An increase in the surface-to-volume ratio with the decreasing size of organic structures leads to the possibility of efficient inactivation of viruses and bacteria under low-dose and non-harmful-visible pulsed light irradiation. Hence, this method can be applied in many fields, such as the instantaneous inactivation of pathogenic viruses and bacteria in a safe and simple manner without damaging large organic structures.

## Introduction

Inactivation with ultraviolet (UV) radiation is a well-established technique that has been used widely, including in the purification of water^1–5^, room decontamination^6,7^, and air purification. The wavelength of UV radiation used is generally shorter than 280 nm, which places it in the UVC region (the wavelength region from 250 to 280 nm is typically used); this wavelength is selected because UVC radiation inactivates pathogenic bacteria, viruses and other microorganisms^8–10^. Inactivation is believed to occur via the formation of thymine dimers in deoxyribonucleic acid (DNA) by the absorption of UVC photons; the dimers prevent further replication of the DNA strains^11–13^. However, it is generally known that many types of viruses and bacteria are resistant to UVC radiation, for example, blood-borne pathogens such as human immunodeficiency virus (HIV)^14–16^. Moreover, UVC is strongly absorbed by human cells and protein components; therefore, it raises concerns about damaging plasma components^17^ and causing platelet aggregation^18^. Thus, inactivation with UVC radiation lacks safety when applied in irradiation to the human body to inactivate pathogenic bacteria, viruses and other microorganisms attached to the skin or inside the human body.

To avoid the above problems related to the human body, many alternative methods have been studied such as inactivation by using cold plasma^19,20^, far-UVC light (200–220 nm region)^21–23^, and plasmonic effects^24,25^. However, these methods are still based on high-energy photons or plasma jets, and their effects on the human body have not yet been clarified. On the other hand, inactivation using continuous wave (CW) mode-locked femtosecond (fs: 10^−15^ s) lasers has attracted special interest as a potential alternative to UV irradiation^26–28^ because this method is based on low-energy photons in the visible or near-infrared region (400–800 nm). The inactivation mechanism is reported as impulsive stimulated Raman scattering of an ultrashort fs visible/near-infrared (NIR) laser pulse. The fs laser pulse coherently excites the mechanical vibrations^29,30^ of the protein capsid of target viral particles, leading to damage and inactivation of a broad spectrum of viruses and bacteria^26–28^ without using toxic or carcinogenic chemicals. This method seems to offer minimal concern of adverse effects to the human body^31^. However, fs laser inactivation methods have the following disadvantages: (1) a fs laser system is very expensive and cannot be easily obtained by everybody, (2) the inactivation efficiency is low thus it requires a long treatment time of more than 1 h^26–28^ for inactivation, and (3) it requires an extremely high peak power of the fs pulse on the order of 100 MW/cm^2^ for the inactivation of micrometer-sized bacteria^32^. These features impede the scalability and practical implementation of this photonic inactivation process.

In this work, we demonstrated the efficient inactivation of micrometer-sized bacteria fused with a dye by using a low-power and easily available nanosecond (ns) visible pulse laser (532 nm). We obtained 3-log inactivation of *Escherichia coli* (*E. coli*) bacteria in a short period of treatment time, i.e., on the order of 10 min. The inactivation mechanism obtained here is based on a transient photothermal evaporation effect of *E. coli* bacteria. For example, stained *E. coli* bacteria instantaneously absorbing 10 ns pulse irradiation were photothermally evaporated within 10 ms. This inactivation occurred under transient nonequilibrium states, which is entirely different from the usual photothermal inactivation performed under equilibrium states using CW lasers^33–40^. The combination of transient pulse irradiation and chromophore-fused target viruses and/or bacteria might not damage or heat large organic structures, such as human blood cells and stem cells. Hence, this method can be applied in many fields, such as the instantaneous inactivation of pathogenic viruses and bacteria in a safe and simple manner.

## Materials and methods

### Culturing, staining and enumeration of microorganisms

**Figure 1:**
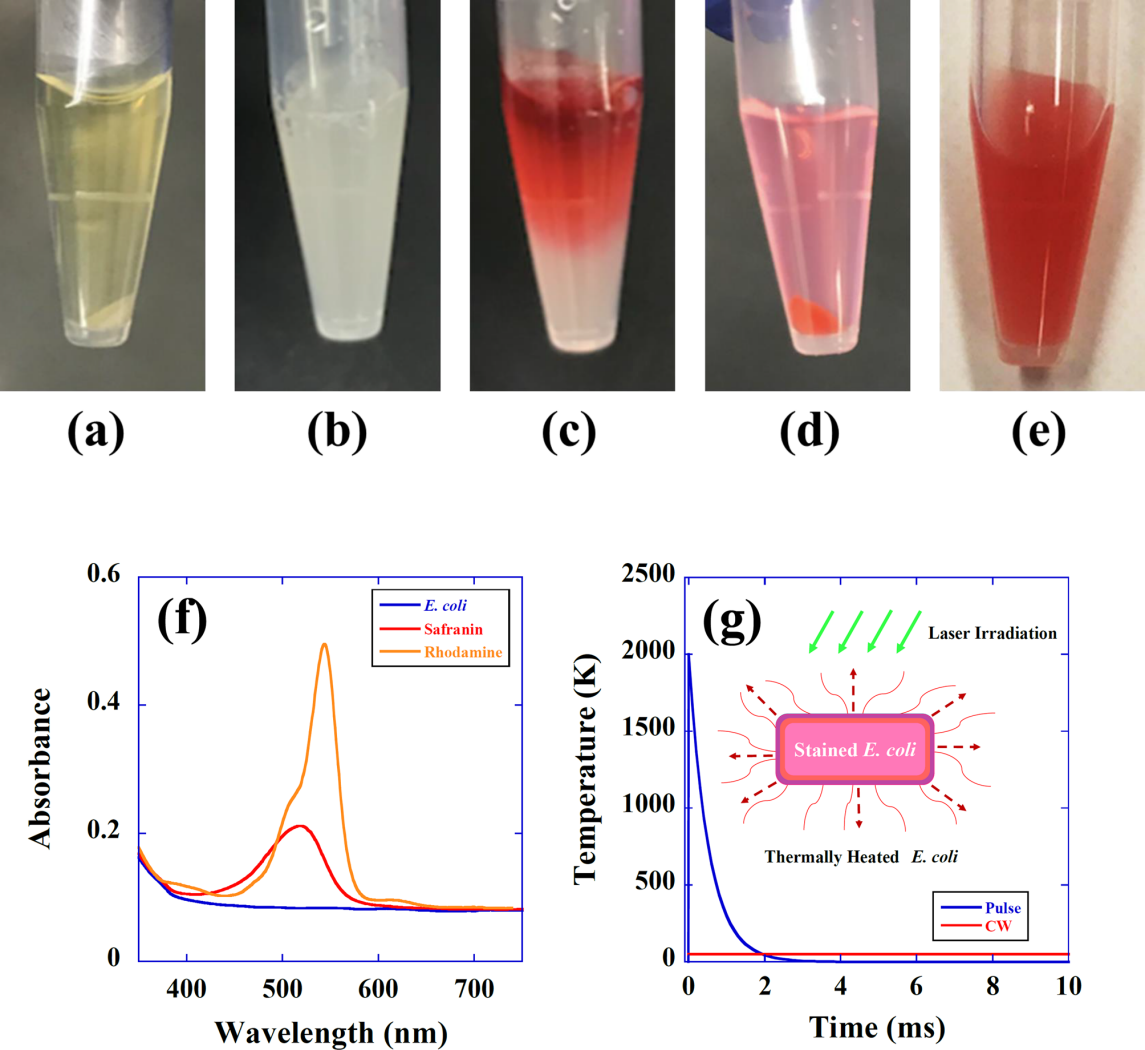
Staining of *E. coli* with a safranin dye solution. (**a**) An *E. coli* suspension (0.5 mL) separated into the supernatant and bacterial cells by centrifugation. (**b**) *E. coli* cells were taken, and 0.5 mL normal saline solution was added to the cells. (**c**) *E. coli* cells in saline solution were stained with a droplet of safranin dye solution. (**d**) Stained cells and safranin dye solution were separated by centrifugation. (**e**) The stained bacterial cells were dissolved in saline solution at a density of 10^4^ CFU/mL. (**f**) Optical absorption spectrum (absorbance) of an unstained *E. coli* solution (blue line), a safranin dye-stained *E. coli* solution (red line), and a rhodamine B dye-stained *E. coli* solution (orange line). The absorbance of *E. coli* at 532 nm was 0.084 for an unstained sample, 0.2 for a safranin-stained sample, and 0.38 for a rhodamine B-stained sample. (**g**) Theoretical plot of a temperature rise of a dye-stained *E. coli* as a function of time for pulsed (blue line) or CW (red line) laser irradiation. Inset shows schematic diagram of the thermal inactivation of *E. coli* by laser irradiation (green arrows), where the laser beam is efficiently absorbed by safranin- or rhodamine B dye-stained *E. coli*. Energy dissipation due to thermal convection is indicated by dashed arrows.

A pure culture of *E. coli* strain DH5α was incubated in nutrient broth (E-MC63; EIKEN Chemical Co., Tokyo, Japan) at 37 °C for 20 h. A concentration of 10^9^–10^11^ colony forming units (CFU)/mL was achieved and used for the experiments. As shown in Fig. 1a, 0.5 mL of *E. coli* suspension was centrifuged at 4000 rpm for 5 min to separate the solution and bacterial cells. The supernatant was removed, and 0.5 mL normal saline solution was added to the cells (Fig. 1b). Then, the cells in saline solution were stained with a droplet of safranin dye solution (30 µL, Hayashi Pure Chemical Industry Limited Corporation, Japan), as shown in Fig. 1c. The stained cells and safranin dye solution were then separated by centrifugation (Fig. 1d). The stained bacterial cells were dissolved in saline solution at a density of 10^4^ CFU/mL, as shown in Fig. 1e. *E. coli* stained with rhodamine B dye (Hayashi Pure Chemical Industry Limited Corporation, Japan) were also produced in the same manner. It should be noted that the reduction behavior (aging) of stained *E. coli* is almost the same as that of unstained *E. coli*; for example, both stained and unstained *E. coli* showed an approximately 10% reduction in CFU after one hour of experiments.

Figure 1f shows the optical absorption spectrum (absorbance) of the *E. coli* solutions stained with safranin dye (red line), rhodamine B dye (orange line) and without any dye (blue line). The absorbance of *E. coli* at the laser excitation wavelength (532 nm) was 0.084 for the unstained sample, 0.2 for the safranin-stained sample, and 0.38 for the rhodamine B-stained sample. Therefore, as shown in Fig. 1g*, E. coli* stained with safranin or rhodamine B dye efficiently absorbed 532 nm laser radiation from the second harmonics (SH) of the yttrium aluminum garnet (YAG) laser, increasing the temperature of *E. coli*. In this case, when a high-intensity laser pulse with the duration of 10 ns irradiates the *E. coli*, the temperature instantaneously increases beyond the evaporation point as shown by the blue line (theoretical plot) of Fig. 1g, leading to destruction of cell structure. On the other hand, with the same dose using CW laser irradiation, instantaneous thermal heating does not occur as shown by the red line (theoretical plot) of Fig. 1g. Thus, pulse irradiation seems to be promising for achieving a much higher inactivation rate than that obtained by CW irradiation at the same dose. We note here that the temperature increase of *E. coli* solution at steady state is the same magnitude between CW and pulsed laser irradiations, because we put the same energy into the solution. The detail of the thermal heating mechanism is described in the discussion section. To perform the inactivation experiments by using the SH of the YAG laser, 600 µL of the stained bacterial cells was taken. Colonies were counted after incubation for 24 h at 37 °C. Plates yielding 1–1000 CFU were considered for analysis. All experiments were performed at least three times independently.

### Inactivation by SH of CW or Q-switched pulsed YAG laser

**Figure 2:**
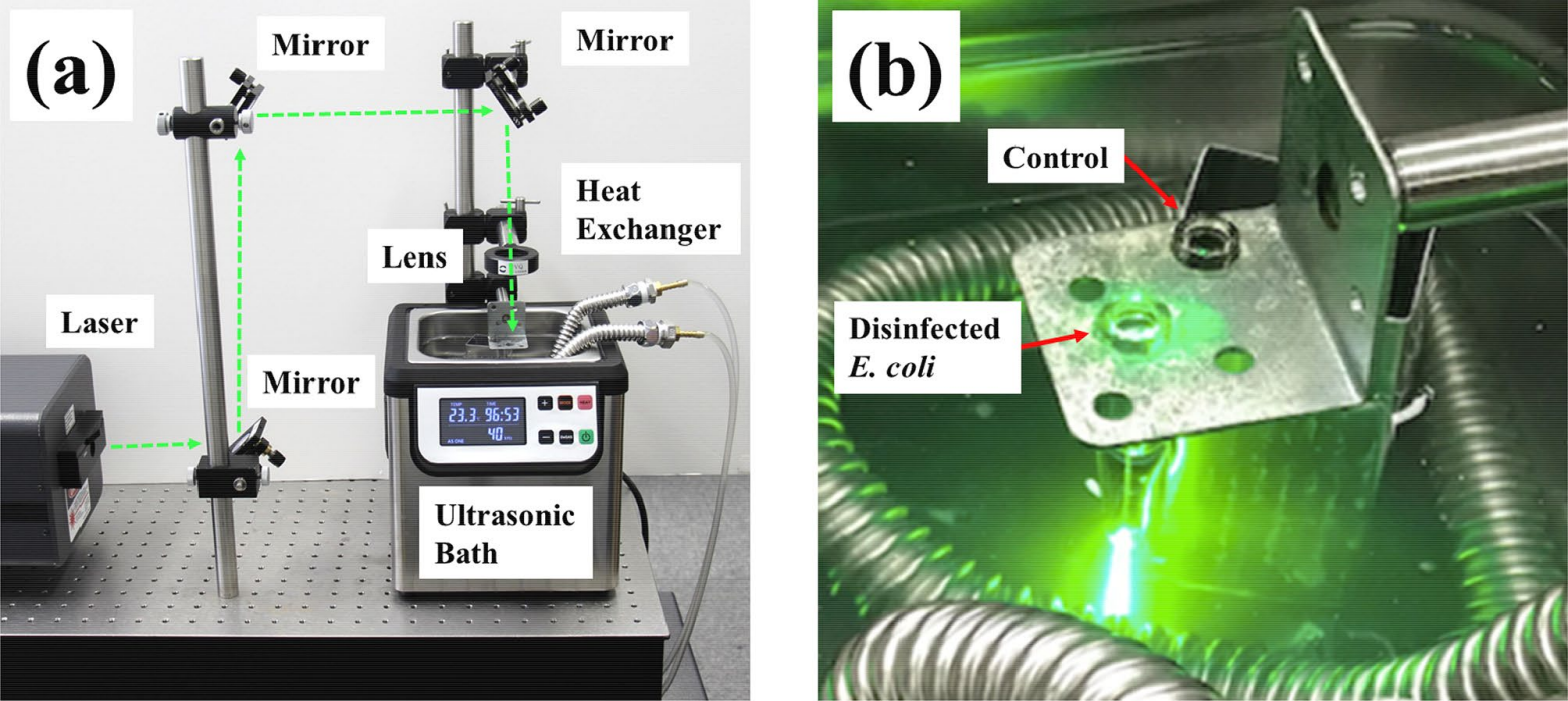
(**a**) Optical setup of the visible laser inactivation system. (**b**) Photograph of an *E. coli* bacterial sample irradiated by the second harmonics of a YAG laser in an ultrasonic bath. An *E. coli* bacterial sample without laser irradiation was also placed in the ultrasonic bath as a control sample to take into account the inactivation caused by ultrasonic vibrations.

Figure 2a shows the optical setup for the inactivation system by using the SH of a CW (100 mW) or pulsed YAG laser (pulse duration: 10 ns, repetition rate: 10 Hz, pulse energy: 10 mJ). A laser beam was guided to a microtube made of borosilicate glass with 5.7 mm φ × 50 mm, which contained a suspension of *E. coli* (600 µL). The focusing beam was made by using a convex lens with a focal length of 200 mm, and the center of the suspension was irradiated by the SH of the YAG laser, as shown in Fig. 2b. The power of both the CW and pulsed YAG laser beams was maintained at 100 mW, and the corresponding power density was 50 W/cm^2^. The inactivation reaction occurred at the central spot of the glass microtube, where the diameter of the focused beam was approximately 0.5 mm, and the irradiated region was approximately 4 µL (0.5φ × 20 mm). The suspension in the tube was homogeneously diffused by using an ultrasonic bath with a frequency of 46 kHz. The temperature of the ultrasonic bath was maintained at 23 °C by using a heat exchanger, where the heat exchanger played a role in inhibiting a temperature increase due to 60 min of ultrasonic operation. (We note here that the temperature of the microtube will rise to approximately 50 °C for 60 min of ultrasonic operation without the heat exchanger). The control suspension, which was not subjected to laser irradiation, was also placed in the ultrasonic bath to precisely distinguish the inactivation caused by the ultrasonic effect from that of laser irradiation. However, it should be noted that the CFU reduction by the ultrasonic treatment was less than 10% of the CFU of the initial control sample; therefore, we used the *E. coli* CFU from the suspension in the ultrasonic bath as the control sample.

## Results

### Inactivation of unstained *E. coli* by CW or pulsed laser treatments

**Figure 3:**
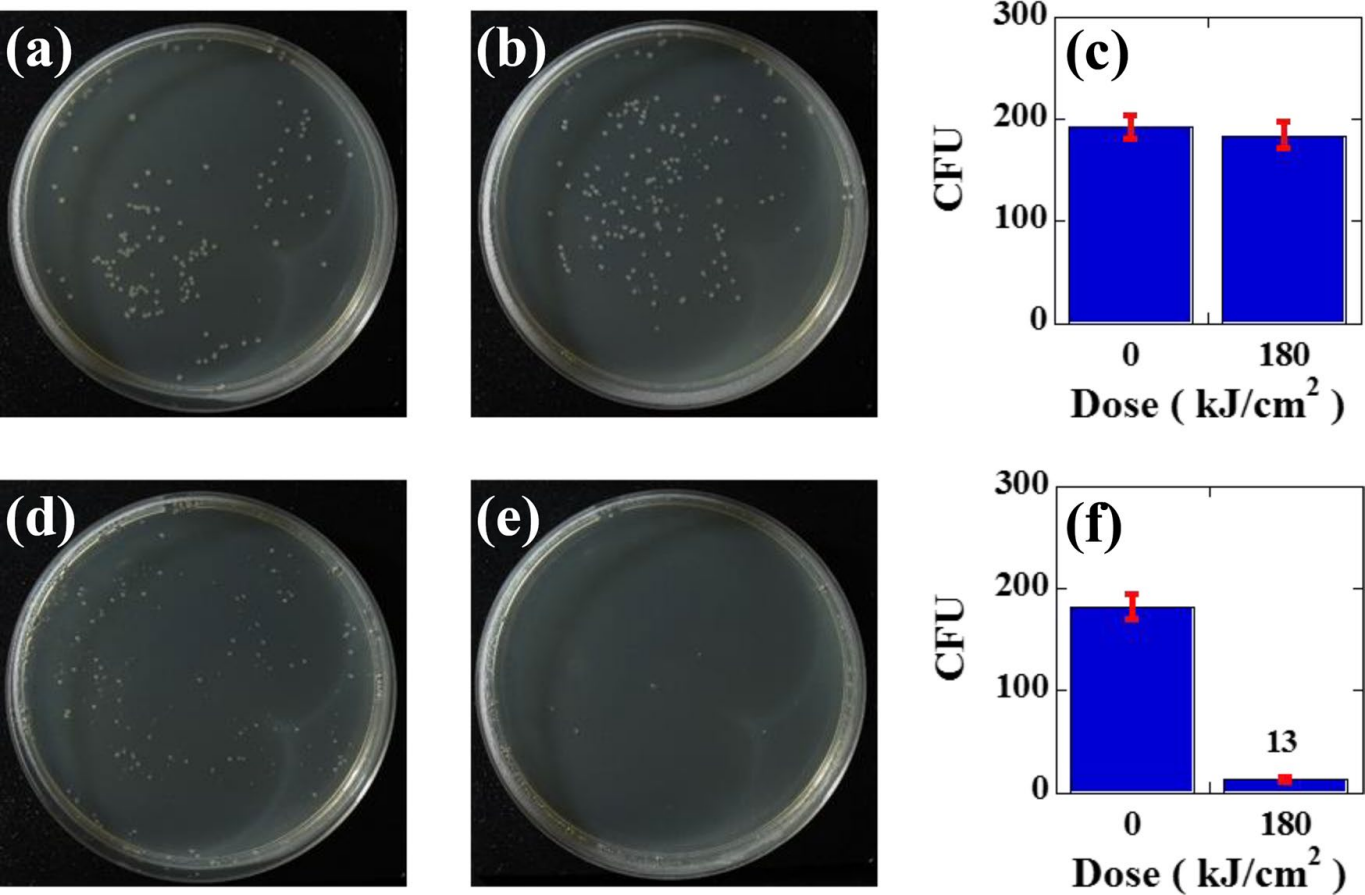
The results of the efficacy of inactivation by using the SH of a CW or Q-switched pulsed YAG laser; (**a**) control plate for the CW YAG laser experiment, (**b**) inactivated plate subjected to 180 kJ/cm^2^ from a 532 nm CW YAG laser, and (**c**) the number of CFU on the control plate (192 ± 11 CFU) and treated plate (184 ± 13 CFU) after treatment with a 532 nm CW YAG laser. (**d**) Control plate of the 532 nm pulsed YAG laser, (**e**) inactivated plate subjected to 180 kJ/cm^2^ from a 532 nm pulsed YAG laser, and (**f**) the number of CFU on the control plate (182 ± 12 CFU) and treated plate (13 ± 2 CFU) after treatment with a 532 nm pulsed YAG laser.

The results of the efficacy of inactivation by using the SH of the CW- or Q-switched pulsed YAG laser system are shown in Fig. 3a–f, where Fig. 3a is the control plate (without CW laser irradiation) of the CW YAG laser experiments and Fig. 3d is the control plate (without pulsed laser irradiation) of the pulsed YAG laser experiments. Figure 3b is the inactivated plate subjected to a 180 kJ/cm^2^ dose (50 W/cm^2^ and 1 h irradiation) from the SH of the CW YAG laser, and Fig. 3e is the inactivated plate subjected to the same dose but using the SH of the pulsed YAG laser. Despite the use of an identical dose, inactivation by pulse irradiation was considerably higher compared to that observed for CW operation. For example, the number of colonies was not reduced by using the CW laser, which was 192 ± 11 CFU (Fig. 3a) for the control plate and 184 ± 13 CFU (Fig. 3b) for that subjected to the 180 kJ/cm^2^ dose of the CW YAG laser. However, when the same dose was implemented with a pulsed laser, the number of colonies was reduced from 182 ± 12 CFU (Fig. 3d) for the control plate to 13 ± 2 CFU (Fig. 3e). The CFU reductions for the CW or pulsed laser experiments are shown in the bar graph of Fig. 3c,f, respectively. Here, error bars for the graphs were calculated and plotted based on the standard deviation. These results clearly showed that a transient heat reaction occurred in *E. coli* due to instantaneous pulse irradiation, whereas thermal heating did not occur with CW irradiation. Thus, pulse irradiation seems to be promising for achieving a much higher inactivation rate than that obtained by CW irradiation at the same dose. However, inactivation caused by visible pulse laser irradiation was not sufficient or effective. This was because of the low absorbance of the bacteria, as shown in Fig. 1f.

### Inactivation of stained *E. coli* by CW or pulsed laser treatments

**Figure 4:**
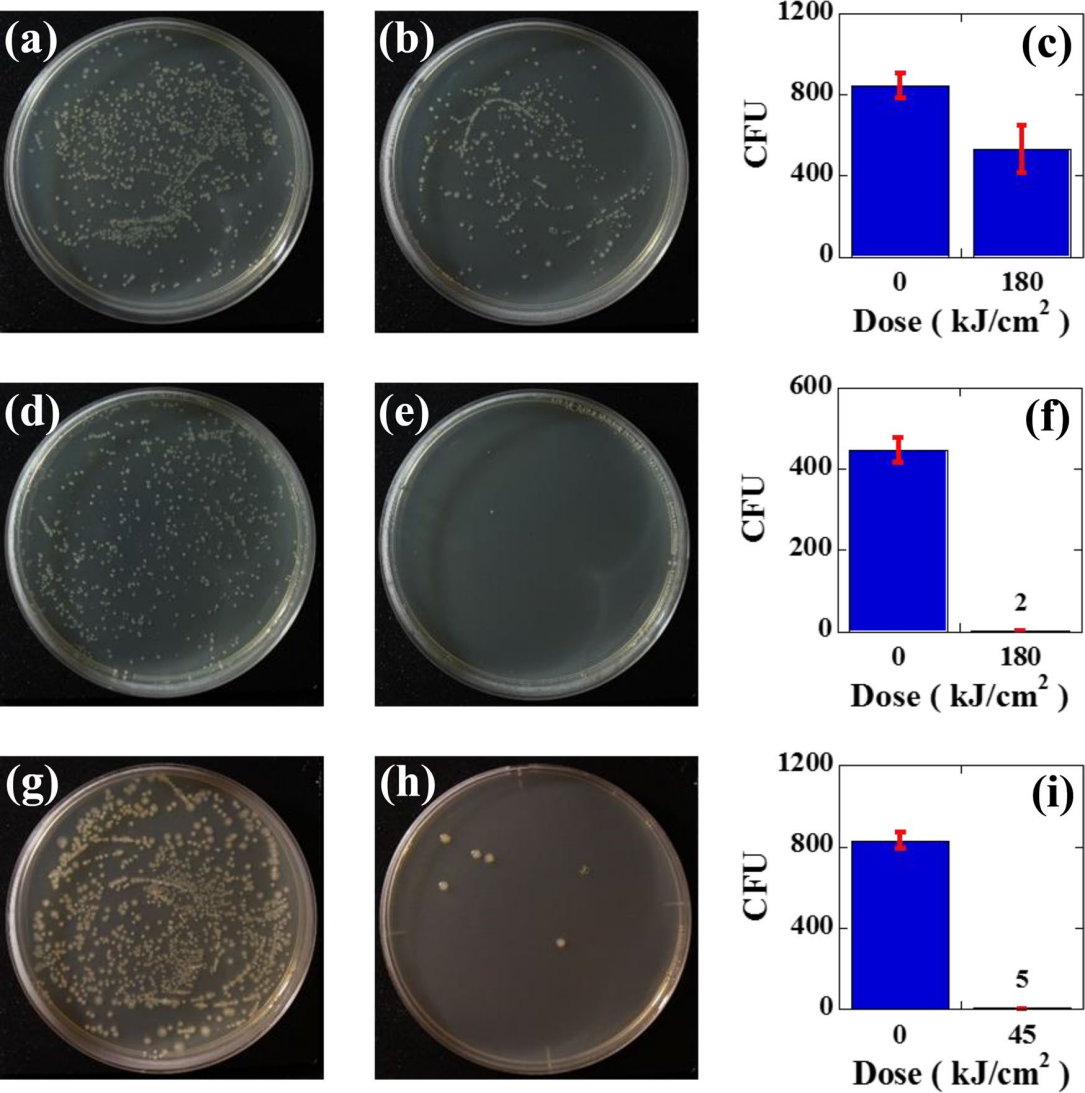
The results of the efficacy of inactivation for safranin-stained *E. coli* obtained by CW YAG laser irradiation: (**a**) control plate, (**b**) inactivated plate subjected to 180 kJ/cm^2^ irradiation, and (**c**) a bar graph showing the number of CFU for the control plate (845 ± 61 CFU) and inactivated plate (530 ± 116 CFU). The results of the efficacy of inactivation for safranin-stained *E. coli* obtained by pulsed YAG laser irradiation; (**d**) control plate, (**e**) inactivated plate subjected to 180 kJ/cm^2^ irradiation, and (**f**) a bar graph showing the number of CFU on the control plate (446 ± 30 CFU) and inactivated plate (2 ± 1 CFU). The results of the efficacy of inactivation for rhodamine B dye-stained *E. coli* obtained by pulsed YAG laser; (**g**) control plate, (**h**) inactivated plate subjected to 45 kJ/cm^2^ irradiation, and (**i**) a bar graph showing the number of CFU for the control plate (831 ± 39 CFU) and inactivated plate (5 ± 1.1 CFU).

The results of the efficacy of inactivation by using the SH of a CW or Q-switched pulsed YAG laser for *E. coli* stained with safranin dye (Fig. 4a–f) and rhodamine B dye (Fig. 4g–i) are shown in Fig. 4, where Fig. 4a–c were obtained with the CW YAG laser, and Fig. 4d–i were obtained with the pulsed YAG laser. When we used the CW laser, we did not observe a significant reduction in the CFU after staining treatment when comparing Fig. 4a with Fig. 4b; for example, the number of colonies was not reduced by using the CW laser, 845 ± 61 CFU for the control plate and 530 ± 116 CFU for the 180 kJ/cm^2^ dose given by a CW YAG laser, as shown in the bar graph of Fig. 4c. However, by applying the same dose with a pulsed laser, reductions in CFU were clearly observed for both safranin- (Fig. 4d,e) and rhodamine B-dyed *E. coli* (Fig. 4g,h). Furthermore, it was clearly observed that the reduction behaviors were strongly correlated with the magnitude of absorbance. For example, the number of colonies was reduced from 446 ± 30 CFU to 2 ± 1 CFU for safranin-dyed *E. coli* (OD: 0.2) with a 180 kJ/cm^2^ dose, as shown in the bar graph of Fig. 4f, and the relatively large absorbance of the rhodamine B-dyed *E. coli* (OD: 0.38) showed a relatively large inactivation rate. For example, almost the same CFU reduction was obtained with the relatively low dose of 45 kJ/cm^2^ for the rhodamine B-dyed sample, such as the reduction from 831 ± 39 CFU to 5 ± 1.1 CFU, as shown in the bar graph of Fig. 4i. Thus, it was clearly shown that the inactivation rate was correlated with the magnitudes of both the absorbance and the irradiation dose.

### Inactivation rate constants of stained or unstained *E. coli* by CW or pulsed laser treatments

**Figure 5:**
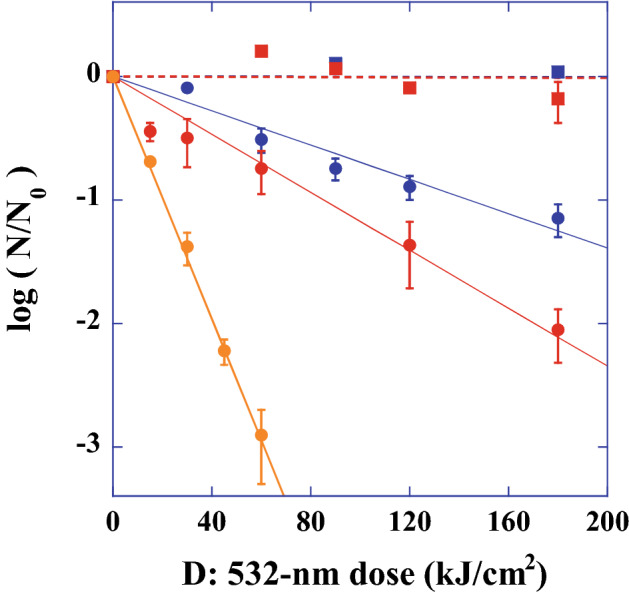
532 nm dose response of stained or unstained *E. coli* inactivated by CW or pulsed YAG laser treatment. Blue squares are the inactivation rates of unstained *E. coli* obtained by CW laser treatment, red squares are the inactivation rates of safranin dye-stained *E. coli* obtained with CW laser treatment, blue circles are the inactivation rates of unstained *E. coli* obtained with pulsed laser treatment, red circles are the inactivation rates of safranin dye-stained *E. coli* obtained with pulsed laser treatment, and orange circles are the inactivation rates of rhodamine B dye-stained *E. coli* obtained with pulsed laser treatment. The dose (D)-based inactivation rate constant κ (cm^2^/kJ) obtained for the pulsed laser was determined by − κD = log_10_[N(D)/N_0_], and κ = 6.95 × 10^−3^ for unstained *E. coli* (solid blue line), κ = 1.17 × 10^−2^ for safranin-stained *E. coli* (solid red line), and κ = 4.91 × 10^−2^ for rhodamine B-stained *E. coli* (solid orange line).

To quantitatively investigate the reduction in inactivation rates as a function of the 532 nm laser irradiation dose, we plotted the CFU response to the 532 nm laser irradiation dose (inactivated CFU by 532 nm irradiation, N(D)), divided by the control CFU (N_0_), caused by the CW laser or pulsed laser treatments, as shown in Fig. 5. Here, D is the magnitude of the dose (kJ/cm^2^), N_0_ is the number of CFU in the unirradiated control (CFU/mL), and N(D) is the number of CFU at a given irradiation dose D. Blue squares are the inactivation rates of unstained *E. coli* obtained with CW laser treatment, red squares are the rates of inactivation of safranin dye-stained *E. coli* obtained with CW laser treatment, blue circles are the rates of inactivation of unstained *E. coli* obtained with pulsed laser treatment, red circles are the rates of inactivation of safranin dye-stained *E. coli* obtained with pulsed laser treatment, and orange circles are the rates of inactivation of rhodamine B dye-stained *E. coli* obtained with pulsed laser treatment. It was clearly observed that CW laser irradiation did not reduce the CFU; that is, the inactivation rate constant was almost equal to 0 for both unstained- or stained *E. coli*, which are denoted by dashed blue or red lines shown in Fig. 5, respectively, while pulsed laser irradiation at the same dose showed a significant reduction in the CFU. Furthermore, an increase in the absorbance (from 0.1 for unstained *E. coli* to 0.38 for rhodamine B-stained *E. coli*) by staining exhibited an additional reduction rate of CFU to the pulsed laser inactivation rates. Based on the above experimentally observed inactivation rates as a function of dose D (kJ/cm^2^), the dose-based inactivation rate constant κ (cm^2^/kJ) determined by − κD = log_10_[N(D)/N_0_] for each sample was obtained by the least square fitting method. The experimentally observed rates were fitted by κ = 6.95 × 10^−3^ for unstained *E. coli* (blue line), κ = 1.17 × 10^−2^ for safranin-stained *E. coli* (red line), and κ = 4.91 × 10^−2^ for rhodamine B-stained *E. coli* (orange line), as shown by the solid lines in Fig. 5. These results are summarized in Table 1. Based on the obtained rate constant κ (cm^2^/kJ), an inactivation efficiency of the pulsed laser irradiation region (the inactivation efficiency inside the beam passing region) η (s^−1^) can be estimated using the following relation, η = κI_0_(V_0_/V), and the efficiency becomes approximately 40%/s for *E. coli* stained rhodamine B dye, 9%/s for safranin-stained *E. coli*, and 5%/s for unstained *E. coli*, where we use I_0_ = 50 W/cm^2^, V_0_ = 4 µL, and V = 600 µL, respectively. The detail of the estimation is given in the following discussion section.

**Table 1:** Inactivation rate constants κ (cm^2^/kJ) obtained experimentally (first line) or theoretically (second line) for unstained, safranin-stained, and rhodamine B-stained *E. coli* irradiated by a CW or pulsed YAG laser.

Inactivation rate constant	Non-stained and pulse	Safranin and pulse	Rhodamine B and pulse	Non-stained and CW	Safranin and CW
κ (Experiment)	6.95 × 10^−3^	1.17 × 10^−2^	4.91 × 10^−2^	0	0
κ (Theory)	6.51 × 10^−3^	1.30 × 10^−2^	5.65 × 10^−2^	3.42 × 10^−5^	6.84 × 10^−5^

## Discussion

Based on the model described in Fig. 1g, we evaluated the temperature increase of a single *E. coli* cell caused by CW or pulsed laser irradiation based on the following heat transfer equation^41^:

1 $$\rho cv\frac{\partial }{\partial t}\left( {T - T_{0} } \right) = \alpha SI\left( t \right) - \gamma S\left( {T - T_{0} } \right) - \varepsilon \sigma S\left( {T^{4} - T_{0}^{4} } \right) ,$$

where *σ* = 5.67 × 10^−12^ (J/s cm^2^ K^4^) is the Stefan-Boltzmann constant^41^, *ρ* is the density (g/cm^3^), *c* is the specific heat (J/g K), *v* is the volume (cm^3^), *T* is the time-dependent temperature due to the absorption of CW or pulsed laser radiation (K), *T*_*0*_ is the temperature before irradiation (K), *α* is the absorption ratio of laser radiation (dimensionless), *S* is the surface (cm^2^), *γ* is the convective heat transfer coefficient (J/s cm^2^ K), and *ε* is the emissivity (dimensionless), of *E. coli*, respectively. In Eq. (1), *I*(t) is the laser-intensity-pulse-profile as a function of time (J/s cm^2^); for CW excitation, *I*(t) takes a constant value as *I*(t) = *I*_*0*_, and for pulse excitation, *I*(t) is expressed, using the Dirac delta function, as *I*(t) = *E*_*p*_δ(t), where *E*_*p*_ is the pulse energy (J/cm^2^) and the delta function has the dimension of s^−1^. As a form of energy dissipation from *E. coli*, we ignored the thermal radiation heat transfer component [*εσS*(*T*^4^ − *T*_0_^4^)] due to its contribution being more than 10 times smaller than that of convection loss [γ*S*(*T* − *T*_0_)] at the considered temperature (*T* = 1000 K, and *T*_0_ = 300 K). Because the energy dissipation terms can be calculated as (i) the convection term, *γS*(*T* − *T*_0_) = 5.6 × 10^−6^ W and (ii) the thermal radiation term, *εσS*(*T*^4^ − *T*_*0*_^4^) = 4.5 × 10^−7^ W, where we uses *S* = 8 × 10^−8^ cm^2^, *γ* = 0.1 (J/s cm^2^ K) obtained in the CW laser experiments [see Eq. (2)], and the maximum emissivity *ε* = 1 is assumed.

For CW irradiation, Eq. (1) can be solved as a steady-state equation. In this case, the temperature increase of the *E. coli* by laser irradiation was given by the following simple equation:

2 $$\left( {T - T_{0} } \right) = \frac{\alpha }{\gamma }I_{0} .$$

Here, *α* could be determined from the absorbance spectrum shown in Fig. 1f and the Beer–Lambert law^42^, and the result was *α* = 2.0 × 10^−3^ for an unstained *E. coli*, *α* = 4.0 × 10^−3^ for a safranin-stained *E. coli*, and *α* = 1.8 × 10^−2^ for a rhodamine B-stained *E. coli*. When the irradiation intensity of the CW laser was 50 W/cm^2^, the temperature of the safranin-stained *E. coli* solution was measured by a thermocouple and increased to 2 °C from the base temperature (*T*_0_). Therefore, by using Eq. (2), the corresponding convective heat transfer coefficient *γ* was determined to be 0.1 (J/s cm^2^ K). The magnitude of this convective heat transfer coefficient was large; therefore, the temperature of the bacterial cell could not be increased by CW excitation.

On the other hand, for pulse irradiation, the transient response of Eq. (1) can be solved by putting *I*(t) = *E*_*p*_δ(t) into it as:

3 $$\left( {T - T_{0} } \right) = \frac{\alpha S}{{\rho cv}}E_{p} exp \left( { - \gamma St/\rho cv} \right) ,$$

and the maximum temperature of *E. coli* can be determined by the absorption ratio (*α*), pulse energy (*E*_*p*_), and surface-to-volume ratio (*S*/*v*). By utilizing the parameters of *E. coli*, such as *ρ* = 1.0 g/cm^3^^38^, *c* = 4.2 J/g K (Here we assume that *c* is equal to the value of water because almost 80% of cells are water^43^), *v* = 1 × 10^−12^ cm^3^, *S* = 8 × 10^−8^ cm^2^, and *E*_*p*_ = 5 J/cm^2^, the theoretically calculated maximum temperature (*T* − *T*_0_)_M_ and the time constant of heat dissipation could be obtained. The temperatures [(*T* − *T*_0_)_M_] were 190 K for unstained *E. coli*, 380 Κ for safranin-stained *E. coli*, and 1710 K for rhodamine B-stained *E. coli*. The time constant γ*S*/ρ*cv* for these samples was 1.9 × 10^3^ Hz. These results are summarized in Table 2. (We note here that the theoretical plot of the temperature rise by pulsed irradiation (blue line) shown in Fig. 1g was obtained by *α* = 2.1 × 10^−2^).

**Table 2:** Theoretically calculated values of the surface-to-volume ratio (*S/v*), absorption ratio (*α*), temperature increase (*T* − *T*_0_), and time constant (*γS/ρcv*) for viruses, bacteria (unstained-, safranin-stained and rhodamine B-stained *E. coli*), and human red blood cells.

	Virus	*E. coli* unstained	*E. coli* safranin	*E. coli* rhodamine	Red blood cells
*S*/*v* (cm^−1^)	1.5 × 10^6^	8 × 10^4^	8 × 10^4^	8 × 10^4^	2 × 10^4^
*α*	0.001 (650 nm)	0.002	0.004	0.018	0.001 (650 nm)
Temp. increase (K)	1780	190	380	1710	24
Time constant (Hz)	36,000	1900	1900	1900	475

To obtain a quantitative comparison, the laser intensity was assumed to be 50 W/cm^2^; repetition rate, 10 Hz; density 1.0 g/cm^3^; and specific heat, 4.2 J/g K, for all the organic structures.

It was clearly seen that the inactivation rate constant κ (cm^2^/kJ) obtained with the results shown in Fig. 5 exhibited a correlation with the maximum temperature (*T* − *T*_0_)_M_, with the linearly proportional relation κ = (*T* − *T*_0_)_M_/ξ, where the parameter ξ (kJ K/cm^2^) is characterized by the temperature and dose of inactivation. When we assumed the parameter ξ = 12.7 × 10^3^ (kJ K/cm^2^), we could evaluate the theoretical values of κ. The calculated values were κ = 6.51 × 10^−3^ for unstained, κ = 1.30 × 10^−2^ for safranin-stained, and κ = 5.65 × 10^−2^ for rhodamine B-stained *E. coli*. The results are summarized in Table 1. The theoretically obtained inactivation rate constants for unstained, safranin dye-stained, and rhodamine B dye-stained *E. coli* agreed well with the experimentally obtained constants. This agreement strongly suggested that the mechanism of inactivation obtained by visible pulse laser irradiation originated from the transient photothermal evaporation effect, which is quantitatively described by κ = (*T* − *T*_0_)_M_/ξ.

It should be noted that due to the factor of the surface-to-volume ratio (*S*/*v*) in Eq. (3), it is possible for relatively small organic structures, such as viruses, to undergo transient photothermal inactivation without any staining treatments. For example, when we irradiated human red blood cells (HRBCs) and viruses at the same time, the wavelength of the pulsed laser was selected to be in a low-absorption region for HRBCs, such as 600 nm or more^44,45^, and the other conditions, such as the density and the specific heat, were assumed to be the same as described above (almost 80% of HRBCs are water^46,47^), the temperature of the HRBCs did not increase and remained at approximately 50 K. On the other hand, the temperature of viruses with structures on the order of 10–100 nm significantly increased up to 1800 K.

**Figure 6:**
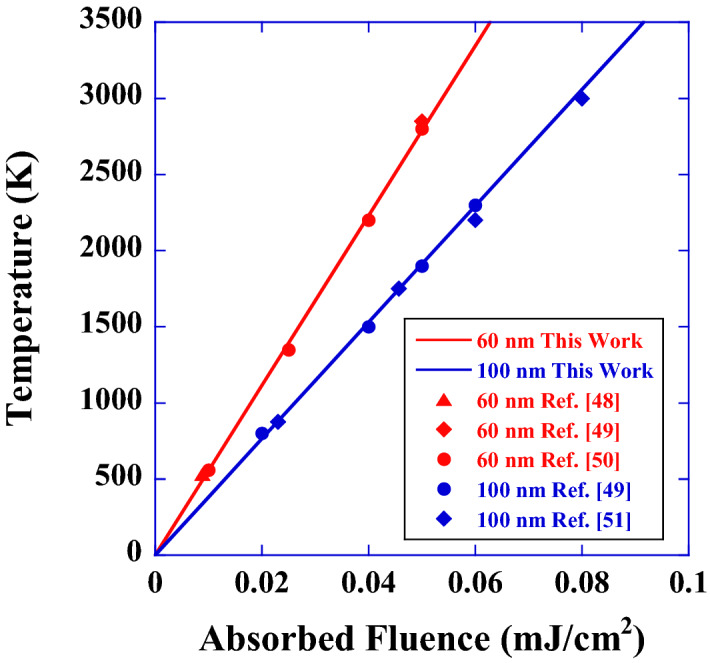
Maximum temperature of a single Au nanoparticle versus absorbed fluence of a pulsed laser. Red and blue lines are the theoretically calculated temperatures of Au nanoparticles with diameters of 60 nm (red line) and 100 nm (blue line). The solid red triangle is the temperature obtained by Ref.^48^, the solid red rhombus is the temperature obtained by Ref.^49^, the solid red circles are the temperatures obtained by Ref.^50^, the solid blue circles are the temperatures obtained by Ref.^49^, and the solid blue rhombi are the temperatures obtained by Ref.^51^. The theoretically calculated maximum temperatures obtained by Eq. (3) agree well with these previously reported temperature values.

It is difficult to directly measure the temperature of the above small organic structures. However, this analysis can be applied not only to small organic structures but also to inorganic nanoparticles whose thermodynamic and thermophysical properties are well established. Here, we quantitatively analyse the temperature of gold (Au) nanoparticles by using Eq. (3) and compare the results with those obtained in previous studies^48–51^. The red and blue lines in Fig. 6 show the theoretically calculated maximum temperatures of Au nanoparticles with diameters of 60 nm (red line) and 100 nm (blue line) as a function of absorbed laser fluence (mJ/cm^2^). To obtain these lines, we used the density of Au nanoparticles of *ρ* = 19.3 g/cm^3^^52^ and the specific heat of *c* = 0.13 J/g K^52^. The theoretically calculated maximum temperatures obtained here coincide with the previously reported values of temperature^48–52^; therefore, we consider that the temperature behaviour of the small organic structures also obeys Eq. (3).

The theoretically calculated values of the surface-to-volume ratio, maximum temperature, absorption ratio and time constant as a function of the size of organic structures are given and compared in Table 2. We considered that short-pulse irradiation yielded an effective and simple method for the inactivation of small pathogens, such as viruses and bacteria.

## Conclusions

In this study, we demonstrated the efficient inactivation of *E. coli* stained with safranin or rhodamine B dyes by using a low-power and easily available nanosecond visible pulse laser and obtained 3-log inactivation of *E. coli* in a short period of treatment time, on the order of 10 min, with a relatively low irradiation dose on the order of 50 kJ/cm^2^. The treatment time and dose magnitude was much faster and much lower, respectively, than those obtained with a fs laser^31,32^.

We used a staining treatment for the inactivation of *E. coli* because, as shown in Fig. 1f*, E. coli* has no absorption band in the visible region. However, there are many bacteria that have inherent absorption bands in the visible region, such as *Pseudomonas aeruginosa* (*P. aeruginosa*), *Staphylococcus aureus* (*S. aureus*), *Micrococcus luteus* (*M. luteus*), and *Kocuria oceani* (*K. oceani*)^53–56^. We consider that by choosing the wavelength of pulsed laser to match the absorption bands, the transient photothermal inactivation of these pathogenic bacteria can be made without the staining treatment. We have some promising results confirming the excitation wavelength dependence of the inactivation efficacy for *M. luteus* and *K. oceani*. These results will be reported elsewhere.

A qualitative model based on the transient photothermal evaporation effect was discussed, and a quantitative evaluation of the temperature increase based on the heat transfer equation was made. As a result of this theoretical analysis, the maximum temperature of bacteria or viruses was correlated with the absorption ratio (*α*), pulse energy (*E*_*p*_), and surface-to-volume ratio (*S*/*v*). The importance of the surface-to-volume ratio leads to the selectivity of inactivation of viruses and bacteria without damaging or heating large organic structures, such as human blood cells and stem cells. We consider that the proposed transient photothermal evaporation method can be applied to many fields such as a sterilization technique of skin and intraoral organ in a convenient manner. To confirm the validity of this method to these practical applications, it is necessary to evaluate the threshold temperature to induce the photothermal evaporation effect for various bacteria and viruses under practical conditions as well as to reduce the irradiation pulse energy.
